# Recent progress in immune‐based interventions to prevent HIV‐1 transmission to children

**DOI:** 10.1002/jia2.25038

**Published:** 2017-12-28

**Authors:** Yegor Voronin, Ilesh Jani, Barney S Graham, Coleen K Cunningham, Lynne M Mofenson, Philippa M Musoke, Sallie R Permar, Gabriella Scarlatti

**Affiliations:** ^1^ Global HIV Vaccine Enterprise New York NY USA; ^2^ Instituto Nacional de Saúde Maputo Mozambique; ^3^ Vaccine Research Center National Institute of Allergy and Infectious Diseases National Institutes of Health Bethesda MD USA; ^4^ Duke University Durham NC USA; ^5^ Elizabeth Glaser Pediatric AIDS Foundation Washington DC USA; ^6^ Makerere University‐Johns Hopkins University Research Collaboration Kampala Uganda; ^7^ Department of Pediatrics and Human Vaccine Institute Duke University Medical Center Durham NC USA

**Keywords:** mother‐to‐child transmission, passive immunization, paediatric vaccine, adolescents, antibodies, HIV

## Abstract

Globally, 150,000 new paediatric human immunodeficiency virus type 1 (HIV‐1) infections occurred in 2015. There remain complex challenges to the global elimination of paediatric HIV‐1 infection. Thus, for the global community to achieve elimination of new paediatric HIV‐1 infections, innovative approaches need to be explored. Immune‐based approaches to prevention of mother‐to‐child transmission (MTCT) may help fill some of the remaining gaps and provide new opportunities to achieve an AIDS‐free generation. Immune‐based interventions to prevent MTCT of HIV‐1 may include paediatric HIV vaccines and passive immunization approaches. Recent discoveries providing evidence of robust immune responses to HIV in infants open new and exciting prospects for paediatric HIV vaccines. Moreover, successful vaccination of infants has a different set of requirements than vaccination of adults and may be easier to achieve. Proof‐of‐concept has been established over the last two decades that passively administered HIV‐1 Env‐specific monoclonal antibody (mAbs) can prevent chimeric simian human immunodeficiency virus (SHIV) transmission to newborn nonhuman primates. There has been tremendous progress in isolating and characterizing broadly neutralizing antibodies to HIV, and clinical testing of these antibodies for treatment and prevention in both infants and adults is a major effort in the field. Immune‐based interventions need to be actively explored as they can provide critically important tools to address persistent challenges in MTCT prevention. It is a pivotal time for the field with active discussions on the best strategy to further reduce HIV infection of infants and accomplish the World Health Organization Fast‐Track 2030 goals to eliminate new paediatric HIV infections.

## 1 Challenges in preventing mother‐to‐child HIV‐1 transmission

Globally, 150,000 new paediatric human immunodeficiency virus type 1 (HIV‐1) infections occurred in 2015 1. This number represents a remarkable 70% decrease since the year 2000. In recent years, the adoption of universal life‐long antiretroviral therapy (ART) for all HIV‐1‐infected pregnant women had a major impact on reducing mother‐to‐child transmission (MTCT) rates in low‐ and middle‐income countries (LMIC). Ambitious goals have been set for 2030 to eliminate new paediatric HIV‐1 infections 2. However, MTCT rates range from 2.0% to 20.6% in the 21 Global Plan countries in Africa, with only five of these countries achieving the World Health Organization (WHO) target of MTCT rates of less than 5%. Over half of transmission occurs postpartum during breastfeeding 1.

Challenges to the global elimination of paediatric HIV‐1 infection include continued acute HIV‐1 infection among women of child‐bearing age, particularly adolescent and young women; high rates of unplanned pregnancies in HIV‐infected women; late presentation to antenatal care; postpartum loss to follow‐up; difficulty maintaining prolonged adherence to ART and viral suppression; and continued transmission through breastfeeding 3. Addressing these challenges will require innovative approaches to re‐shape primary health care delivery and better integrate HIV‐1 care in the overall healthcare services.

Between 2009 and 2015, there has been only a 5% decline in new HIV‐1 infections among women of reproductive age globally 1. In sub‐Saharan Africa, three of every four newly infected adolescents are female 4. Compared to older women, pregnant adolescents and young women <24 years of age are less likely to access antenatal care, undergo HIV‐1 testing, and receive ART; consequently, MTCT rates in this population are significantly higher than among older mothers 5, 6. Incident HIV‐1 infection during pregnancy and breastfeeding continues to be a significant problem, with rates in some areas of sub‐Saharan Africa as high as 3% to 5% annually, similar to that seen in high risk “key populations” 7. MTCT is two to three times higher among pregnant and breastfeeding women with incident versus chronic HIV‐1 infection 7.

Equitable access to family planning services, comprehensive antenatal care and institutional deliveries remain low in many sub‐Saharan Africa countries, and combined with sustained rates of incident infection among young women, continued MTCT can be expected due to successive cohorts of newly infected women becoming pregnant 6. Over a third of pregnant women in LMIC first initiate antenatal care in the third trimester or have no antenatal care, which limits the efficacy of ART in preventing MTCT. Delayed initiation of ART in pregnancy is associated with higher levels of maternal HIV‐1 viraemia at delivery and higher MTCT rates 8.

Globally, 80% of pregnant women identified as HIV‐1‐infected were receiving antiretroviral drugs for either prophylaxis or treatment in 2015, but ranges from less than 20% in Middle‐East and North‐Africa to around 90% in East‐ and Southern‐Africa 1. However, in some countries, adherence to and retention in life‐long ART antenatal/postpartum programs have been relatively poor 9, 10, 11. Even among women achieving initial viral suppression on ART, rebound viraemia occurs in up to one‐third, particularly during the postpartum period, increasing the risk of postnatal transmission 12. Ongoing viral replication has also been observed in a significant minority of women who conceive while receiving ART 13, 14.

While current approaches to prevention of MTCT have had an important impact in averting new paediatric HIV‐1 infection, the success in LMIC has been limited by weaknesses of health systems and by programme‐level challenges. These challenges have not been mitigated by implementing a simplified approach of universal ART for pregnant/breastfeeding women (Option B+). For example, comparing the antenatal cascade in over 28,000 women presenting to antenatal care in Malawi before and after implementing Option B+ in 2011, although a greater proportion of HIV‐1‐infected pregnant women were enrolled in services and initiated ART, there were significant incremental losses after ART initiation 15. A recent assessment of 18‐month MTCT rates in South Africa under universal maternal ART found overall 18‐month transmission was 4.1%, with most transmission occurring postpartum 16. Thus, for the global community to achieve elimination of new paediatric HIV‐1 infections, new approaches need to be explored. In this article, we describe how immune‐based approaches to prevention of MTCT may help fill some of the remaining gaps and provide new opportunities to achieve an AIDS‐free generation.

While scale‐up of current ART interventions for prevention is a priority, the addition of immune‐based interventions can assist in overcoming a number of challenges that have been observed to date 17. A significant problem has been difficulties with adherence to daily ART, particularly postpartum, leading to rebound viraemia. For the mother‐infant pair, the time of delivery is the most consistent time of contact with the healthcare system and a single subcutaneous injection of potent monoclonal antibody (mAbs) to the neonate at birth would provide long‐acting protection (up to six months), addressing the problems of access and adherence. HIV‐1 active immunization of the infant, who is already being seen frequently for the routine childhood immunizations, could provide long‐lasting protection against breast milk transmission for the infant and may potentially persists into adolescence and beyond. Furthermore, an effective immune‐based intervention can be delivered universally, which would protect infants whose mothers become infected while breastfeeding, a population that is not protected with current strategies.

## 2 Prospects for a paediatric HIV‐1 vaccine

Developing a preventive HIV‐1 vaccine is an enormous challenge that so far has met with only limited success in the RV144 trial in Thailand 18. The follow‐up trial to RV144 began in 2016, while another vaccine candidate entered efficacy testing in December of 2017. These trials will be conducted in adults, and no plans have been announced to test these candidates in infants. There are at least three arguments that can be made for accelerated testing of candidate HIV‐1 vaccines in children.

First, preventive HIV‐1 vaccines will ultimately need to be implemented as part of a routine immunization schedule prior to sexual debut. Including HIV‐1 immunization in the childhood vaccine schedule provides the opportunity for high coverage with a multi‐dose vaccine, as well as long boosting intervals to improve the likelihood of affinity maturation for antibodies. Multi‐dose vaccine strategies have been difficult to implement outside the childhood immunization schedule. For example, immunization coverage for hepatitis B virus (HBV), which is both a sexually and perinatally transmitted pathogen, significantly improved when it was moved from an adolescent vaccine to a neonatal vaccine 19. When efficacy of a candidate HIV‐1 vaccine is demonstrated, advancing the product into children and preadolescents will be substantially accelerated if safety and immunogenicity data are already available, including information on onset and durability of effective immunity.

The infant immune system is often described as immature, but it is more accurate to say that it has a different set of regulatory constraints and capacities. The immune responses of infants and young children are often more robust than adults and may have a broader unmodified repertoire of T‐cells and B‐cells that could more effectively be primed by vaccination 20. Recent studies of HIV‐infected infants show that they develop neutralizing activity more rapidly than adults, simultaneously targeting multiple sites of vulnerability on HIV‐1 envelope, and broadly neutralizing monoclonal antibodies (bNAbs) isolated from HIV‐1‐infected infants have substantially lower somatic hypermutation rates than typically seen in bNAbs isolated from adults 21. In addition, HIV‐1‐exposed infants vaccinated with MF59/recombinant gp120 HIV‐1 vaccine had higher magnitude and more durable anti‐V1V2 IgG2 responses than adults immunized with the same vaccine 22. Moreover, studies have established for HBV, human papilloma virus (HPV), and HIV‐1 that the infant response to subunit vaccines can be of higher magnitude compared to those of adults 23. Therefore, it would be prudent to take advantage of the immunological benefits of vaccinating children to address the remaining HIV‐1 MTCT.

**Figure 1:**
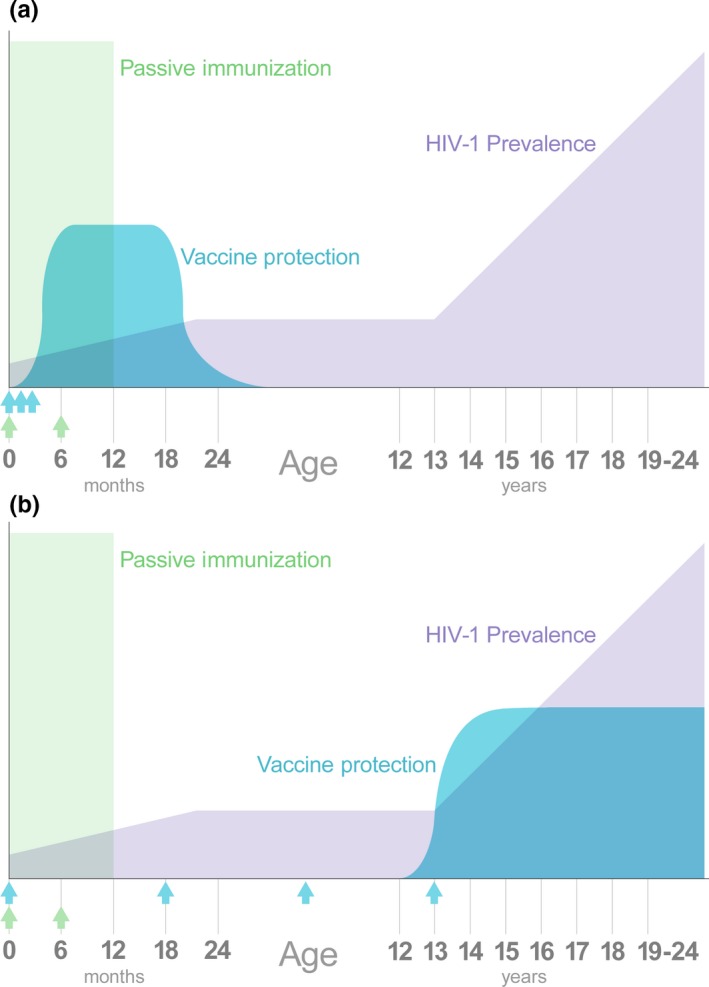
Unique opportunities for immune‐based intervention in children and adolescents. In the absence of interventions, HIV‐1 infections in children are biphasic, with high risk at birth and during breastfeeding, a pause during most of childhood, and then increasing risk with the onset of sexual maturity. Initiating passive immunization immediately after birth (green arrows) would provide an independent mechanism of protection to complement existing interventions. (**a**) **A vaccine administered immediately after birth (blue arrows) may be appropriate for the infant population during the breastfeeding period, even if its duration of protection is short. (b) The prolonged period of low HIV‐1 risk provides an opportunity to administer a vaccine that requires multiple spaced‐out doses (blue arrows).**

Finally, successful vaccination of infants has a different set of requirements than vaccination of adults and may be easier to achieve (Figure 1). For example, a vaccine that induces protective immunity for only 12‐18 months may not be suitable to address the adult HIV‐1 epidemic, but would make a large impact in preventing HIV‐1 infection during breastfeeding.

## 3 Advances in passive immunotherapy

Initial studies of HIV‐1 hyperimmune globulin in HIV‐1‐infected pregnant women and/or their newborns utilized polyclonal preparations of highly purified human immune globulin containing high titres of antibody to HIV‐1 structural proteins that did not have significant anti‐viral activity, required frequent (monthly) administration, and did not demonstrate efficacy for prevention in studies in the US and Uganda 24, 25.

However, the identification of human anti‐HIV‐1 mAbs with high potency and neutralizing breadth, combined with protein engineering to extend half‐life and optimize potency, and advances in mAb manufacturing have made the use of mAbs in prevention of MTCT feasible and practical. In a recent review of potential use of monoclonal antibodies for prevention of sexual HIV‐1 transmission, Anderson and colleagues note that with optimization of antibody manufacturing platforms, bnAbs will be available at a significantly reduced cost in the near future; using their estimated target cost of $10/gram product, the cost for an infant dose of 100 mg could be as low as $1.00 26. Cost‐effectiveness studies will be useful to evaluate the cost‐benefit of the addition of an immune‐based HIV‐1 prevention intervention in infancy.

Proof‐of‐concept has been established over the last two decades that passively administered HIV‐1 Env‐specific mAbs can prevent chimeric simian human immunodeficiency viruses (SHIV) transmission to newborn nonhuman primates 27, 28, 29. Hessel *et al*. provided additional evidence to support the value of passively administering bNAbs to newborns to prevent HIV‐1 infection 30. Newborn macaques treated with two bNAbs (VRC07 and PGT121) given subcutaneously beginning 24 hours after SHIV‐exposure demonstrated prevention and clearance of infection even from sites of initial replication. Phase I and II clinical trials with second‐generation CD4 binding‐site antibodies have demonstrated safety in adults and infants 31. Efficacy evaluation of VRC01 bNAb is underway in high‐risk men and women in North and South America and Southern Africa, and will provide proof‐of‐concept for whether neutralizing antibodies can prevent HIV‐1 infection of humans.

**Figure 2:**
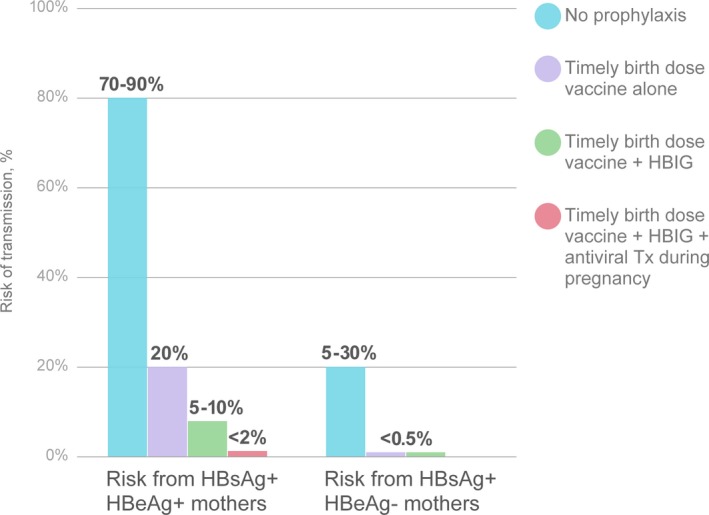
Combination of drug‐ and immune‐based approaches allows drastic reduction in rates of hepatitis B virus transmission from mothers to children.

The precedent for administering polyclonal and monoclonal antibodies to newborn infants for the prevention of viral disease has been established for HBV and respiratory syncytial virus (RSV), respectively. Indeed, MTCT of HBV at birth has been nearly eliminated in developed countries by administration of birth‐dose intramuscular hepatitis B immunoglobulin and vaccination (Figure 2) 32. However, active‐passive immunization strategy is less effective in mothers who are HBV e‐antigen positive and have high levels of HBV viraemia, resulting in higher rates of transmission than in HBV e‐antigen negative mothers. In this situation, addition of the anti‐viral drug tenofovir to active‐passive immunization has reduced HBV transmission to near zero, demonstrating the benefits of combining different prevention strategies 33, 34, 35. For HIV‐1, the combination of maternal ART with passive‐active prophylaxis can provide immediate protection to the infant during the intrapartum and early breast‐feeding period should there be viraemia due to late presentation and/or non‐optimal ART, rebound viraemia due to adherence problems, or maternal infection with antiretroviral‐drug resistant virus; passive immunization can be followed by active immunization to provide longer‐lasting protection should maternal ART adherence be intermittent during the breastfeeding period.

Palivizumab for RSV has been marketed since 1998 and can safely and effectively reduce the frequency of serious RSV disease in high‐risk infants. New generation RSV mAbs with higher potency and extended half‐lives are in the final stages of efficacy evaluation and are being developed for use as a single dose at birth. These examples, together with the data from animal models and the advances in development and manufacturing, suggest that administration of mAbs to newborns at risk of HIV‐1 infection would be an effective and practical adjunct to antiretroviral treatment to prevent MTCT of HIV‐1 at birth and through breastfeeding. This may be especially useful in high prevalence areas with high infection pressure, in situations where there has been limited access to prenatal care, or when HIV‐1 in the mother is diagnosed late.

An open‐label, dose‐escalating phase 1 study of safety and pharmacokinetics of VRC01 delivered subcutaneously at birth (P1112 study) is ongoing in babies born to HIV‐1 infected women in USA, South Africa and Zimbabwe 36. To date, the product has been safe with a half‐life of approximately 20 days. A birth dose of 40 mg/kg results in a day 28 level above 50 μg/ml in almost all infants 31. The half‐life of VRC01 has now been extended by Fc modification (VRC01‐LS) to increase binding to neonatal Fc‐receptors 37. VRC01‐LS is currently being tested in adults with plans to evaluate it in infants as an additional arm of the P1112 study. The cumulative effects of increased potency, breadth and durability may make it possible to protect infants through at least six months of life with just one or two doses of mAbs. A phase 2b trial in infants would provide the opportunity to confirm safety and assess efficacy against MTCT of HIV‐1 in combination with ARVs and provide a means by which to further lower transmission rates and accomplish the 2030 goal of eliminating paediatric HIV‐1 infections.

Even if one imagines the optimal scenario where 100% of the 1.5 million HIV‐infected women giving birth annually are tested, identified, and initiated on ART prior to the third trimester and transmission rates observed in clinical trials (e.g. PROMISE) are achieved, the residual 1.5% transmission rate would translate to nearly 23,000 newly infected infants born annually. In reality, over 30% of pregnant women do not receive HIV testing; many women in sub‐Saharan Africa only present for antenatal care in the third trimester or at delivery 38, and in this population, as well as women who have persistent or rebound viraemia at delivery and postpartum, transmission rates to infants are likely to be closer to 5%, similar to that observed in adult prevention studies in the era of ART and pre‐exposure prophylaxis, and infants at that level of risk require additional prevention options.

## 4 Summary

The tremendous progress in preventing MTCT of HIV‐1 is tempered by complex remaining health system and societal challenges in many LMIC. In addition to efforts to improve delivery of well‐established interventions, it is important to explore new approaches that may have complementary or even synergistic impact with existing efforts to prevent HIV‐1 transmission to infants 39. Effective vaccine is a long‐term goal, but studies in infants need to be initiated today to accelerate translation of adult vaccines to paediatric population and to take advantage of the unique aspects of HIV‐1 epidemiology and vaccine implementation in children and adolescents. Passive immunization is a rapidly maturing technology that may help address some of the persisting challenges in preventing HIV‐1 MTCT, particularly the remaining risk and challenges of preventing postnatal MTCT in LMIC.

## Competing interests

The authors declare that they have no competing interests.

## Authors’ contributions

YV, IJ, GS wrote the first draft. YV, IJ, BSG, CKC, LMM, PMM, SRP, GS edited the manuscript.
